# Prospective multicenter assessment of patient preferences for properties of gadolinium-based contrast media and their potential socioeconomic impact in a screening breast MRI setting

**DOI:** 10.1007/s00330-021-07982-y

**Published:** 2021-05-28

**Authors:** Sean A. Woolen, Jonathan P. Troost, Shokoufeh Khalatbari, Akshat C. Pujara, Jennifer S. McDonald, Robert J. McDonald, Prasad Shankar, Alana A. Lewin, Amy N. Melsaether, Steven M. Westphal, Katherine H. Patterson, Ashley Nettles, John P. Welby, Parth Pradip Patel, Neud Kiros, Lisa Piccoli, Matthew S. Davenport

**Affiliations:** 1 Department of Radiology and Biomedical Imaging, UCSF, 350 Parnassus, San Francisco, CA 94117 USA; 2 Michigan Institute for Clinical & Health Research, Michigan Medicine, Ann Arbor, MI USA; 3 Department of Radiology and Imaging Sciences, Emory University School of Medicine, Atlanta, GA USA; 4 Department of Radiology, Mayo Clinic, Rochester, MN USA; 5 Department of Radiology, Michigan Medicine, Ann Arbor, MI USA; 6 Michigan Radiology Quality Collaborative, Michigan Medicine, Ann Arbor, MI USA; 7 Department of Radiology, Center for Biomedical Imaging, New York University School of Medicine, New York, NY USA; 8 Department of Radiology, Mount Sinai, New York, NY USA; 9 Department of Radiology and Imaging Sciences, Indiana University School of Medicine, Indianapolis, IN USA

**Keywords:** Magnetic resonance imaging, Mass screening, Gadolinium, Contrast media, Patient-centered care

## Abstract

**Objective:**

It is unknown how patients prioritize gadolinium-based contrast media (GBCM) benefits (detection sensitivity) and risks (reactions, gadolinium retention, cost). The purpose of this study is to measure preferences for properties of GBCM in women at intermediate or high risk of breast cancer undergoing annual screening MRI.

**Methods:**

An institutional reviewed board-approved prospective discrete choice conjoint survey was administered to patients at intermediate or high risk for breast cancer undergoing screening MRI at 4 institutions (July 2018–March 2020). Participants were given 15 tasks and asked to choose which of two hypothetical GBCM they would prefer. GBCMs varied by the following attributes: sensitivity for cancer detection (80–95%), intracranial gadolinium retention (1–100 molecules per 100 million administered), severe allergic-like reaction rate (1–19 per 100,000 administrations), mild allergic-like reaction rate (10–1000 per 100,000 administrations), out-of-pocket cost ($25–$100). Attribute levels were based on published values of existing GBCMs. Hierarchical Bayesian analysis was used to derive attribute “importance.” Preference shares were determined by simulation.

**Results:**

Response (87% [247/284]) and completion (96% [236/247]) rates were excellent. Sensitivity (importance = 44.3%, 95% confidence interval = 42.0–46.7%) was valued more than GBCM-related risks (mild allergic-like reaction risk (19.5%, 17.9–21.1%), severe allergic-like reaction risk (17.0%, 15.8–18.1%), intracranial gadolinium retention (11.6%, 10.5–12.7%), out-of-pocket expense (7.5%, 6.8–8.3%)). Lower income participants placed more importance on cost and less on sensitivity (*p* < 0.01). A simulator is provided that models GBCM preference shares by GBCM attributes and competition.

**Conclusions:**

Patients at intermediate or high risk for breast cancer undergoing MRI screening prioritize cancer detection over GBCM-related risks, and prioritize reaction risks over gadolinium retention.

**Key Points:**

*• Among women undergoing annual breast MRI screening, cancer detection sensitivity (attribute “importance,” 44.3%) was valued more than GBCM-related risks (mild allergic reaction risk 19.5%, severe allergic reaction risk 17.0%, intracranial gadolinium retention 11.6%, out-of-pocket expense 7.5%).*

*• Prospective four-center patient preference data have been incorporated into a GBCM choice simulator that allows users to input GBCM properties and calculate patient preference shares for competitor GBCMs.*

*• Lower-income women placed more importance on out-of-pocket cost and less importance on cancer detection (p < 0.01) when prioritizing GBCM properties.*

**Supplementary Information:**

The online version contains supplementary material available at 10.1007/s00330-021-07982-y.

## Introduction

Selection of an optimal GBCM has gained focus in recent years based on evidence that a tiny quantity of gadolinium administered with GBCM is retained in the brain and body for months or years [1–4]. The clinical importance of such gadolinium retention is unknown, but is a relevant consideration, especially in young patients and those receiving repeated lifetime administrations (e.g., women at intermediate or high risk for breast cancer undergoing MRI screening) [5–7]. However, choice of gadolinium-based contrast media (GBCM) is more complex than gadolinium retention alone and influenced by its potential benefits (e.g., detection sensitivity) as well as its risks (e.g., adverse reaction rate, intracranial gadolinium retention, cost) [8].

It is currently unclear how patients prioritize this recently emphasized [6] gadolinium retention relative to other GBCM risks and benefits [8]. For example, is a GBCM with low risk of long-term gadolinium retention but low detection sensitivity and high allergic-like reaction rate preferred over a GBCM with higher risk of long-term gadolinium retention, higher detection sensitivity, and lower allergic-like reaction rate? The complex interplay between these factors can leave GBCM selection to provider gestalt, and often without patient input. The aim of this study was to measure preferences for properties of GBCM in an annual screening MRI population at greatest potential risk of GBCM-related side effects. We prospectively studied patients at risk for gadolinium retention at four centers to elicit their implied preferences for GBCM properties.

## Material and methods

This Health Insurance Portability and Accountability Act-compliant prospective discrete choice conjoint survey was approved by the Institutional Review Boards at each of 4 participating institutions. Informed consent was obtained from all participants. The Standardizing Reporting of Observational Studies in Epidemiology (STROBE) guidelines were used in the preparation of this manuscript.

### Study population

We conducted a prospective observational discrete choice conjoint survey at 4 institutions in Michigan, Minnesota, New York, and Indiana from July 2018 to March 2020. The institutions were chosen to support a broad range of demographic characteristics. Patients participating in breast MRI screening programs are known to have a skewed demographic distribution compared to the general population [9–13]. The inclusion criteria were chosen to reflect individuals with a vested personal interest in cancer detection and a high likelihood of repeated lifetime administrations of GBCM. Inclusion criteria were as follows: (1) outpatient, (2) intermediate or high risk for breast cancer [14] undergoing annual screening breast MRI. Exclusion criterion was previous participation in the study (N = 0). Indications for MRI were based on the American Cancer Society’s 2007 guidelines for breast cancer screening with MRI [14].

### Conjoint survey development and administration

Our study design used a paired discrete choice-based conjoint survey. This type of survey provides respondents with 2 options and asks them to select which one they would prefer. Each option has a series of attributes that the investigators wish to study. In our context, those attributes were the risks and benefits of GBCM. That process is repeated multiple times. At the conclusion of the survey, implied preferences can be derived that indicate what attributes were prioritized by the respondents when making their choices.

Our discrete choice-based conjoint survey (Supplemental Material; Sawtooth Software, Inc (Provo, UT, USA); [15]) used a partial profile design and provided respondents with 15 paired choice sets. Each choice was between two unique hypothetical GBCM with the same 5 attributes set at the same or different levels (sensitivity for cancer detection [range 80–95%], intracranial gadolinium retention [range 1–100 molecules retained per 100 million molecules administered], severe allergic-like reaction rate [range 1–19 per 100,000 administrations], mild allergic-like reaction rate [range 10–1000 per 100,000 administrations], and out-of-pocket cost [range $25–$100]). Only one answer was allowed per question. The range of most GBCM attribute levels was derived from the literature (cancer detection sensitivity [16, 17], gadolinium retention [18], severe allergic-like reaction rate [19], and mild allergic-like reaction rate) [19]. Out-of-pocket cost was informed by common co-pay rates, and online drug prices and proprietary vendor-negotiated price contracts from participating institutions. The content of the survey was vetted by patient advocates with experience in survey design and underwent precognitive pilot-testing for content and readability by five patients undergoing breast MRI who were not included in the study and who met inclusion criteria. A professional medical illustrator created infographic information to facilitate patient understanding.

The survey software considered the active ratings of the respondents to generate a personalized choice set that maximized analyzability of their responses. The experiment had a near-orthogonal design with level balance and minimal attribute level overlap. Details of the administered surveys are provided in Supplementary Table 1.

The survey was administered by trained interviewers. Potential participants were recruited by reviewing the daily breast MRI schedule at each participating institution and consenting patients in real-time the day of their examination. Demographic data (patient age, patient ethnicity, indication for MRI) were extracted from the electronic medical record for all screened patients at each site to (1) reduce the question burden from each patient, and (2) to characterize the non-respondent population.

### Sample size calculation

An a priori power calculation was performed to estimate the needed sample size. Based on the largest observed standard deviation available from preliminary data, to achieve a ± utility confidence interval length of 10 required 170 patients, and to achieve a ± utility confidence interval length of 5 required 670 patients. The study was terminated after 236 patients had been accrued due to the emerging novel coronavirus disease 2019 (COVID-19) pandemic [20].

### Statistical design

Hierarchical Bayesian modeling and a Monte Carlo Markov chain algorithm were used to estimate part-worth utilities (and their 95% confidence intervals) for each GBCM attribute [21–23]. A total of 50,000 posterior simulation iterations were used. Part-worth utilities were an interval measure of patient preference for levels within an attribute—somewhat analogous to a beta coefficient from a logistic regression. To aid interpretation, part-worth utilities were zero-centered so that positive values indicated increased likelihood of selection and negative values indicated decreased likelihood of selection.

Attribute importance is the estimated average relative importance participants placed on a given attribute when making GBCM selection decisions. For each participant, attribute importance was calculated as the range of their part-worth utilities for that attribute, divided by the sum of the ranges for all attributes, multiplied by 100 (i.e., $\frac{Specific\ attribute\ utility\ range}{\sum All\  attribute\ utility\ ranges}\times 100$). The average attribute importance was reported as a percentage and a 95% confidence interval. Attribute importances summed to 100%.

Demographic data were summarized with descriptive statistics. Patient-specific attribute importances were modeled using linear regression. The covariates used for analysis included age, education, health insurance, employment, household income, and previous allergic-like reaction to GBCM. The mean attribute importance difference was calculated between each covariate subgroup.

Statistical analysis was performed using SAS software (v9.4). For primary endpoints, *p* < 0.05 is considered significant. For secondary endpoints (i.e., when assessing differences in attribute importance), *p* < 0.01 was considered significant to account for multiple comparisons.

### Creating a GBCM preference simulator based on patient preference data

We created a simulator that could be used to ascertain how current and future (i.e., hypothetical) GBCM products would perform in the marketplace (relative to each other) if GBCM selection was solely based on the patient preference data we collected in our multi-site study. Utility values for a combination of GBCM properties were combined to build a multi-product competitive model using the randomized first choice method to estimate share of preference. Patient-specific share of preference for a GBCM was calculated as the antilog of the total product utility (based on patient-specific part-worth utilities). Results for each product were rescaled to sum to 100%. Overall share of preference was calculated as the mean of patient-specific shares of preference. Patient-centered simulations were performed to compare 3 existing GBCM (using published data) with 3 hypothetical GBCM using the patient-level part-worth utilities derived from our study. The specific GBCM property information is included in Table 1. Although these six GBCM (3 existing, 3 hypothetical) were the only GBCM formally analyzed in our simulations, the simulator derived from our data (Supplementary Material) permits the user to input any combination of attribute levels based on existing or novel GBCM to determine its hypothetical value (from the patient’s perspective) vs. a user-defined number of competitor GBCM.

**Table 1:** Gadolinium-based contrast media (GBCM) properties for simulation products

GBCM	Chemical Structure	Sens (%)	Out-of-Pocket Expense ($)	Gadolinium Retention (per 100M molecules)	Severe Reaction Rate (/100k)	Mild Reaction Rate (/100k)
Existing Product A	Linear ionic	83	83	4.5	2.1	39
Existing Product B	Linear ionic	94	100	4.0	12	130
Existing Product C	Macrocyclic	94	72	0.2	5.7	150
Test Product D^(a)^	Linear nonionic	83	25	20	1.6	12
Test Product E^(b)^	Macrocyclic	78	100	0.2	12	72
Test Product F^(b)^	Macrocyclic	83	75	0.1	18	130

^(a)^Test product D is a hypothetical linear non-ionic GBCM with similar breast cancer sensitivity to existing product A, high gadolinium retention, low allergic-like reaction rates, and low out-of-pocket cost

^(b)^Test products E and F are hypothetical macrocyclic GBCM with low breast cancer sensitivity, low intracranial gadolinium retention, intermediate to high allergic-like reaction rates, and intermediate to high out-of-pocket cost

## Results

### Patient characteristics

**Fig. 1:**
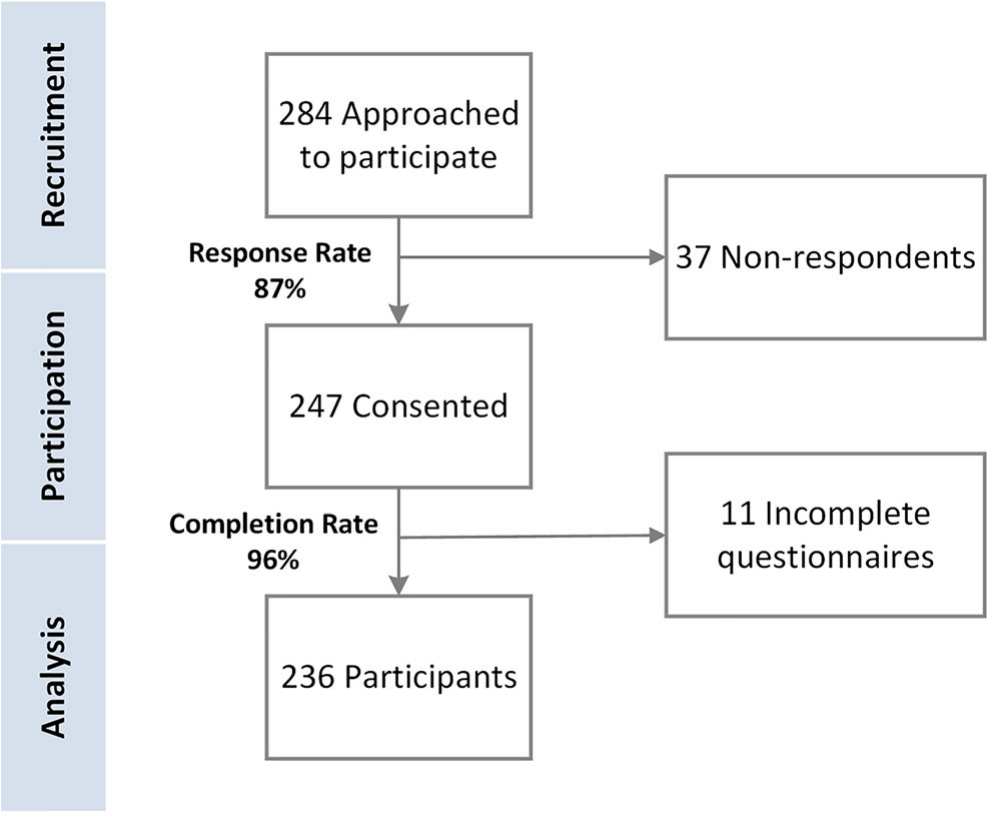
Study population flow diagram

The survey response (87% [247 of 284]) and completion (96% [236 of 247]) rates were excellent. A total of 284 patients were approached to participate; 247 agreed to participate and 236 completed the survey. Incomplete surveys (N = 11) were excluded from analysis. A study population flow diagram is provided in Fig. 1. Non-responders (N = 37) had similar demographics to responders (N = 236) (Table 2). Recruitment distribution from the four sites was as follows: site 1 (38% [90 of 236]); site 2 (30% [70 of 236]); site 3 (16% [38 of 236]); site 4 (16% [38 of 236]). Details of our study population are provided in Table 2.

**Table 2:** Participant characteristics

Characteristic	Responders (n = 236)	Non-responders (n = 37)
Age (years)		
Mean (SD)	50 (11.9)	53 (12.5)
Median (1st quartile to 3rd quartile)	51 (41 to 59)	52 (44 to 61)
Range	26 to 77	27 to 75
Gender, n (%)		
Female	236 (100)	37 (100)
Race, n (%)		
White	201 (85)	37 (100)
Hispanic	9 (4)	0 (0)
Black	15 (6)	0 (0)
Asian	10 (4)	0 (0)
Other	1 (0)	0 (0)
Breast cancer risk, n (%)		
High-risk mutation (BRCA1, BRCA2, other)	64 (27)	6 (16)
Untested, first-degree relative with high-risk mutation	12 (5)	0 (0)
Chest radiation between 10 and 30 years of age	9 (4)	2 (5)
> 20% lifetime risk of breast cancer	179 (76)	22 (60)
One or more intermediate risk factors	88 (37)	20 (54)
Education, n (%)		
Less than high school	2 (1)	
High school graduate	26 (11)	
Trade/technical/vocational	17 (7)	
Associates degree	26 (11)	
Bachelor degree	74 (31)	
Master or Doctorate degree	90 (38)	
Prefer not to answer	1 (0)	
Health insurance, n (%)		
Self-insured	11 (5)	
Employer-based plan	176 (75)	
Medicaid	12 (5)	
Medicare	22 (9)	
Other	15 (6)	
Employment status, n (%)		
Full-time employment	150 (64)	
Part-time employment	28 (12)	
Disabled	6 (3)	
Unemployed	14 (6)	
Retired	38 (16)	
Household income, n (%)		
Less than $25,000	12 (5)	
$25,000–$49,999	18 (8)	
$50,000–$74,999	16 (7)	
$75,000–$99,999	42 (18)	
$100,000–$149,999	47 (20)	
More than $150,000	69 (29)	
Prefer not to answer	32 (14)	
Previous allergic-like reaction to GBCM, n (%)		
Yes	5 (2)	
No	231 (98)	

A majority (85% [201 of 236]) of participants were white with a mean age of 50 ± 11.9 years. Most had a household income greater than $75,000 (77% [158 of 204] among those reporting income), were college educated (70% [164 of 236]), had full-time employment (64% [150 of 236]), and had an employer-based insurance plan (75% [176 of 236]). Only 2% (5 of 236) reported a previous allergic-like reaction to GBCM.

### Importance of GBCM attributes

**Fig. 2:**
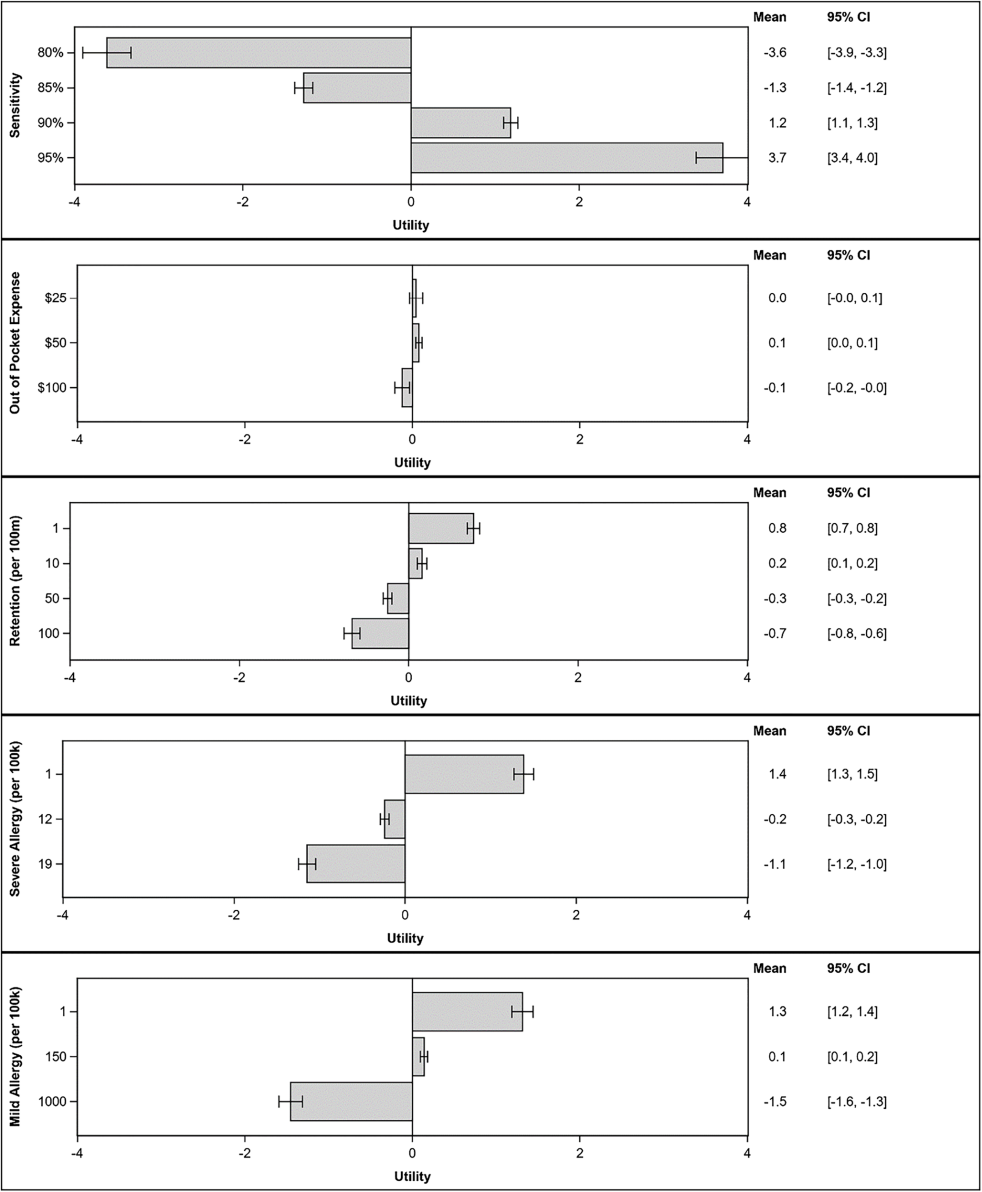
Tornado plots of average part-worth utilities and 95% confidence intervals for all attribute levels

The values respondents ascribed to each GBCM attribute, expressed as part-worth utilities, are included in Fig. 2, with pairwise comparisons between levels provided in Supplementary Table 2. Participants preferred (*p* < 0.001) greater cancer detection sensitivity, lower cost, less intracranial gadolinium retention, and lower mild and severe allergic-like reaction rates (Fig. 2, Supplementary Table 2). Patients considered cancer detection sensitivity to be the most important GBCM attribute (Fig. 2, Table 3). GBCM attributes are listed as follows in descending order of importance (%, Table 3): (1) cancer detection sensitivity (44.3%, 95%CI 42.0–46.7%), (2) mild allergic-like reaction rate (19.5%, 95%CI 17.9–21.1%), (3) severe allergic-like reaction rate (17.0%, 95%CI 15.8–18.1%), (4) intracranial gadolinium retention (11.6%, 95%CI 10.5–12.7%), (5) out-of-pocket cost (7.5%, 95%CI 6.8–8.3%). Intracranial gadolinium retention was considered by patients to be less important than cancer detection sensitivity and allergic-like reaction rates (mild and severe), but more important than out-of-pocket cost.

**Table 3:** Average attribute importance and univariable linear regression results testing differences in mean attribute importance by participant characteristics

Importance	Sensitivity	Out-of-pocket expense	Gadolinium retention	Severe reaction rate	Mild reaction rate
Mean (%) [95% CI]	44.3 [42.0, 46.7]	7.5 [6.8, 8.3]	11.6 [10.5, 12.7]	17.0 [15.8, 18.1]	19.5 [17.9, 21.1]
Income					
< $25k	33.1 [23.0, 43.1]	14.9 [7.1, 22.6]	15.7 [10.6, 20.8]	15.6 [11.8, 19.4]	20.7 [14.4, 27.0]
> $150k	48.8 [45.1, 52.5]	5.7 [4.9, 6.5]	10.1 [8.2, 11.9]	17.7 [15.5, 19.8]	17.8 [15.2, 20.4]
Comparisons: mean importance differences [99% CI]					
Univariable model	Sensitivity	Out-of-pocket expense	Gadolinium retention	Severe reaction rate	Mild reaction rate
Age (/10 years)	−0.9 [−3.5, 1.7]	−0.2 [−1.1, 0.6]	0.0 [−1.2, 1.2]	0.9 [−0.4, 2.2]	0.1 [−1.6, 1.9]
College vs. no college	6.5 [−0.2, 13.2]	−2.8 [−4.9, −0.7]*	−0.9 [−4.0, 2.3]	1.4 [−1.9, 4.7]	−4.2 [−8.8, 0.3]
Employer-based insurance vs. others	1.2 [−5.9, 8.3]	−1.1 [−3.4, 1.2]	−0.2 [−3.5, 3.1]	0.5 [−2.9, 4.0]	−0.4 [−5.2, 4.4]
Full-time work vs. other	−0.4 [−6.9, 6.0]	−1.6 [−3.7, 0.4]	0.1 [−2.9, 3.1]	0.7 [−2.4, 3.9]	1.2 [−3.1, 5.6]
Household income					
< $50k vs. ≥ $50k	−9.1 [−18.3, 0.2]	5.2 [2.3, 8.1]*	5.1 [0.8, 9.3]*	−0.2 [−4.8, 4.4]	−1.1 [−7.4, 5.3]
< $75k vs. ≥ $75k	−7.2 [−15.0, 0.7]	4.7 [2.2, 7.1]*	3.5 [−0.1, 7.2]	−1.0 [−4.9, 2.9]	−0.0 [−5.5, 5.4]
< $100k vs. ≥ $100k	−9.4 [−15.9, −2.9]*	3.5 [1.5, 5.6]*	3.0 [−0.1, 6.0]	−0.7 [−4.0, 2.6]	3.7 [−0.9, 8.2]
< $25k vs. ≥ $150k	−15.7 [−30.2, −1.3]*	9.2 [4.7, 13.7]*	5.7 [−1.2, 12.5]	−2.0 [−9.4, 5.3]	2.9 [−7.1, 12.9]
Prior allergic reaction vs. no	−15.3 [−36.6, 6.0]	−1.8 [−8.7, 5.1]	5.4 [−4.6, 15.3]	5.3 [−5.2, 15.7]	6.5 [−8.1, 21.1]

*p < 0.01. The bold with * emphasizes the values with statistical significance

Attribute “importance” is the estimated average relative importance participants placed on that attribute when making product selection decisions. For each participant, attribute importance (%) is calculated as the range of their part-worth utilities for that attribute, divided by the sum of the ranges for all attributes multiplied by 100 (i.e., $\frac{Specific\ attribute\ utility\ range}{\sum All\  attribute\ utility\ ranges}\times 100$). Reported values in the first row above are the average importance across all 236 participants

Attribute importance varied by several factors including household income and educational attainment (Table 3, Supplementary Table 3). Compared to patients with an annual income > $150,000, patients with an annual income < $25,000 placed less value on cancer detection sensitivity (importance 33.1% vs. 48.8%, *p* < 0.01), greater value on out-of-pocket cost (importance 14.9% vs. 5.7%, *p* < 0.01), and greater value on intracranial gadolinium retention (15.7% vs. 10.1%, *p* < 0.01) (Table 2). College-educated patients placed slightly less importance on out-of-pocket cost (−2.8%, 99%CI −4.9 to −0.7) (Table 3). However, the rank-ordering of attribute importance between these income and educational strata was nearly the same, and cancer detection sensitivity always had the highest importance. Age, insurance status, employment status, and level of breast cancer risk did not have a significant effect on GBCM attribute importance (Table 3). Race and ethnicity subgroups were insufficiently powered for subgroup analysis.

### Preference share simulator model

Multi-product competitive simulations were performed to determine which GBCM a population of patients would prefer if they knew the details and were empowered to choose which GBCM they were administered (Table 4). In all simulations, existing product C (macrocyclic, high sensitivity 94%, low intracranial gadolinium retention 0.2 per 100 million molecules, high mild reaction rate 150/100,000, low severe reaction rate 5.7/100,000, intermediate out-of-pocket cost $72) was the most preferred (preference share 57.4–61.7% competing vs. 2–5 other GBCM) (Table 4). This was driven by superior sensitivity for cancer detection, low gadolinium retention, and low severe reaction rate (Table 3).

**Table 4:** Simulation results of share of preference

Scenario	Sensitivity (%)	Out-of-pocket expense ($)	Gadolinium retention (per 100M molecules)	Severe reaction rate (/100k)	Mild reaction rate (/100k)	Share of preference (%)
Scenario 1						
Existing product A	83	83	4.5	2.1	39	16.5
Existing product B	94	100	4	12	130	21.8
Existing product C	94	72	0.2	5.7	150	61.7
Scenario 2						
Test product D	83	25	20	1.6	12	13.0
Existing product A	83	83	4.5	2.1	39	8.8
Existing product B	94	100	4	12	130	20.3
Existing product C	94	72	0.2	5.7	150	57.8
Scenario 3						
Test product E	78	100	0.2	12	72	2.9
Existing product A	83	83	4.5	2.1	39	14.4
Existing product B	94	100	4	12	130	21.5
Existing product C	94	72	0.2	5.7	150	61.3
Scenario 4						
Test product F	83	75	0.1	18	130	1.6
Existing product A	83	83	4.5	2.1	39	15.7
Existing product B	94	100	4	12	130	21.5
Existing product C	94	72	0.2	5.7	150	61.2
Scenario 5						
Existing product A	83	83	4.5	2.1	39	8.0
Existing product B	94	100	4	12	130	20.1
Existing product C	94	72	0.2	5.7	150	57.4
Test product D	83	25	20	1.6	12	12.0
Test product E	78	100	0.2	12	72	1.6
Test product F	83	75	0.1	18	130	0.9

Hypothetical macrocyclic GBCM (products E and F) with low cancer detection sensitivity (78–83%), low intracranial gadolinium retention (0.1–0.2 per million molecules), and intermediate to high allergic-like reaction rates (mild, 72–130/100,000; severe, 12–18/100,000) were less preferred than two existing linear GBCM (products A and D) with similar cancer detection sensitivity (83%), intermediate to high gadolinium retention (4.5–20 per million molecules), and low allergic-like reaction rates (mild, 12–39/100,000; severe, 1.6–2.1/100,000) (Table 4). Even though the two hypothetical macrocyclic agents had lower intracranial gadolinium retention, the higher patient importance for low mild and severe reaction rates made the linear agents A and D (preference shares: 8.0% and 12.0%) more preferred than macrocyclic agents E and F (preference shares: 1.6% and 0.9%) (Tables 3 and 4).

## Discussion

Patients at intermediate or high risk for breast cancer undergoing screening MRI screening strongly prioritize cancer detection (attribute importance 44.3%) over GBCM-related risks (attribute importance 11.6–19.5%). This is predictable because patients undergo a test when they perceive the benefits outweigh the risks. It also implies that clinically meaningful differences in GBCM relaxivity are likely to be valued by patients. Among GBCM-related risks, patients place greater importance on allergic-like reactions (17.0–19.5%) than gadolinium retention (11.6%), and greater importance on gadolinium retention (11.6%) than out-of-pocket cost (7.5%). These relationships are maintained regardless of patient demographics and background, but the degree of importance patients place on these attributes varies by household income and presence of a college education. We believe these data can be used to inform the selection or innovation of contrast media for patients undergoing repeated lifetime contrast-enhanced MRI—a population potentially at greatest risk of GBCM-related side effects.

GBCM selection from the patient’s perspective is more nuanced than the recent focused attention on gadolinium retention [8]. This point is relevant because newly described potential risks like gadolinium retention sometimes can receive outsized importance in clinical decision-making. In our population, gadolinium retention and cost were less important to patients than allergic-like reaction risks and cancer detection sensitivity. The slightly greater importance patients placed on mild vs. severe reactions likely relates to the ranges of tested prevalence (mild, 10–1000/100,000; severe, 1–19/100,000). The rarity of severe reactions likely counterbalanced their severity. This also illustrates that, from the patient’s perspective, “nuisance” mild reactions have relevance and that relevance is prioritized over the uncertain clinical importance of gadolinium retention. Reaction rates probably should be considered at least as important as (if not more important than) gadolinium retention during GBCM selection.

Despite recruiting from 4 institutions, the demographics of our patient population (white 85%, income > $75,000 77%, college educated 81%, full-time employment 64%, employer-based healthcare 75%) were skewed relative to the US general population [24]. Rather than study-related selection bias, this likely reflects disproportionate access to breast MRI screening in the USA [9–13]. Haas et al (2016) analyzed 316,172 women aged 35–69 years from 5 Breast Cancer Surveillance Consortium registries and found that non-Hispanic white women with < 20% lifetime risk of breast cancer were 62% more likely than non-white women to receive an MRI, and that college-educated women in that cohort were 132% more likely to receive an MRI than those with a high school education or less [9]. In women at high risk (≥ 20% lifetime risk of breast cancer), there was no significant difference in MRI access by race or ethnicity, but high-risk women with no more than a high school education were significantly less likely to receive an MRI than those with a college education (relative risk 0.40) [9].

In our study, household income affected how patients weighted GBCM attributes. In particular, patients with less household income placed greater importance on out-of-pocket cost (+3.5 to +9.2%) and less importance on detection sensitivity (−7.2 to −15.7%). These data reflect how financial pressure affects healthcare decision-making and viewed broadly can contribute to worse clinical outcomes [25–28]. In our study, patients with less income were exchanging diagnostic accuracy for less immediate out-of-pocket cost. Not only were impoverished patients making choices that hypothetically could impair their health, they were less likely to access MRI screening in general. Poverty contributes to poor breast cancer outcomes due to lack of primary care, inadequate health insurance, and poor healthcare access [28]. Finding ways to address the barriers of poverty and other social determinants of health [27] is necessary to attain equity in the US healthcare.

There were several limitations of our study. There was not a pre-existing conjoint instrument for external validation. To address this, we had our instrument reviewed and approved by patient advocates with experience in survey design, used infographics and explanatory text to improve comprehension, and performed precognitive testing for content and readability by five patients prior to dissemination. We intentionally performed our analysis from the patient’s point of view even though patients are not generally involved in choosing a GBCM. This was done to inform radiologists and GBCM vendors which GBCM attributes patients consider most important. We used a breast MRI screening population because this population is exposed to repeated lifetime doses of GBCM, has a vested interested in cancer detection, and is potentially at greatest risk (if any) from long-term gadolinium retention. Our results may be different in other populations (e.g., those receiving a single GBCM dose). Risk of bias in survey administration was minimized by use of professional conjoint software that automates a near-orthogonal design with level balance and minimal attribute level overlap. Sampling bias was minimized by recruiting from 4 institutions and having an excellent response rate (87%).

In conclusion, patients at intermediate or high risk for breast cancer undergoing MRI screening prioritize cancer detection sensitivity over GBCM-related risks, and prioritize reaction risks over gadolinium retention. Although these relationships were consistent across various demographic and socioeconomic strata, patients with less household income were more willing to exchange GBCM diagnostic accuracy for affordability. These data, and the simulator, should be useful when selecting or innovating contrast media for patients undergoing annual MR screening.

## Supplementary Information

ESM 1 (DOCX 27 kb)

ESM 2 (PDF 1681 kb)

ESM 3 (XLSX 187 kb)

## Abbreviations

GBCM: Gadolinium-based contrast media
STROBE: Standardizing Reporting of Observational Studies in Epidemiology

## Declarations

### Guarantor

The scientific guarantor of this publication is Sean Woolen MD MSc.

### Statistics and biometry

The authors Jonathan P Troost PhD and Shokoufeh Khalatbari MS have significant statistical expertise.

### Methodology

• prospective,

• observational,

• multicenter study at 4 institutions.
