# Supplementary material for: Prospective multicenter assessment of patient preferences for properties of gadolinium-based contrast media and their potential socioeconomic impact in a screening breast MRI setting
Source: Eur Radiol. 2021 May 28;31(12):9139–49. doi: 10.1007/s00330-021-07982-y (PMC8160413; doi:10.1007/s00330-021-07982-y)
Supplement: Supplementary file 1 — (DOCX 27 kb) [file 330_2021_7982_MOESM1_ESM.docx]

**Supplementary Table 1. Survey characteristics**

| **Survey Information** | **Frequency** |
| --- | --- |
| # of patients | 236 |
| # of tasks per-patient | 15 |
| # of choices per-task | 2 |
| Total # of patient-tasks | 3540 |
| # of unique profiles tested* | 430 out of 432 possible |
| # of unique comparisons† | 3128 out of 93096 possible |
| **Attribute levels**†† | **n (%)** |
| **Sensitivity, n (%)** |  |
| 8.0 out of 10 (80%) | 1769 (25) |
| 8.5 out of 10 (85%) | 1764 (25) |
| 9.0 out of 10 (90%) | 1761 (25) |
| 9.5 out of 10 (95%) | 1786 (25) |
| **Out of pocket expense, n (%)** |  |
| $25.00 | 2360 (33) |
| $50.00 | 2358 (33) |
| $100.00 | 2362 (33) |
| **Retention, n (%)** |  |
| 1 molecule remains for every 100 million  molecules administered (0.000001%) | 1777 (25) |
| 10 molecules remain for every 100 million molecules administered (0.00001%) | 1777 (25) |
| 50 molecules remain for every 100 million molecules administered (0.00005%) | 1760 (25) |
| 100 molecule remains for every 100 million molecules administered (0.0001%) | 1766 (25) |
| **Severe allergic reactions, n (%)** |  |
| 1 in hundred-thousand (0.001%) | 2356 (33) |
| 12 in hundred-thousand (0.012%) | 2361 (33) |
| 19 in hundred-thousand (0.019%) | 2363 (33) |
| **Mild allergic reactions, n (%)** |  |
| 10 in hundred-thousand (0.01%) | 2366 (33) |
| 150 in hundred-thousand (0.15%) | 2360 (33) |
| 1000 in hundred-thousand (1%) | 2354 (33) |

| *432 possible= 4 (levels of sensitivity) x 3 (levels of cost) x 4 (levels of gadolinium retention) x 3 (severe reaction rates) x 3 (mild reaction rates). 430 of 432 unique combinations of attribute levels were tested at least once.  †93,096 possible= _n_C_r_=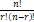=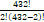. Explicitly testing all possible comparisons of all possible combinations is not feasible.  ††Among all GBCM profiles tested, the distributions of levels within an attribute are fixed to be (approximately) uninform |
| --- |

**Supplementary Table 2. Pairwise comparison of attribute levels, results of linear-mixed effects models**

| **Pairwise level comparison** | **Difference in partworth utilities**  **mean [95% CI]** | **Tukey-adjusted P-value** |
| --- | --- | --- |
| **Sensitivity** |  |  |
| 85% vs. 80% | 2.3 [2.0, 2.7] | <0.001 |
| 90% vs. 80% | 4.8 [4.5, 5.1] | <0.001 |
| 95% vs. 80% | 7.3 [7.0, 7.6] | <0.001 |
| 90% vs. 85% | 2.5 [2.8, 2.1] | <0.001 |
| 95% vs. 85% | 5.0 [5.3, 4.7] | <0.001 |
| 95% vs. 90% | 2.5 [2.8, 2.2] | <0.001 |
| **Out of pocket expense** |  |  |
| $50 vs. $25 | 0.0 [-0.1, 0.1] | 0.78 |
| $100 vs. $25 | -0.2 [-0.3, -0.1] | 0.004 |
| $100 vs. $50 | -0.2 [-0.3, -0.1] | <0.001 |
| **Retention** |  |  |
| 10 per 100m vs. 1 per 100m | -0.6 [-0.7, -0.5] | <0.001 |
| 50 per 100m vs. 1 per 100m | -1.0 [-1.1, -0.9] | <0.001 |
| 100 per 100m vs. 1 per 100m | -1.4 [-1.5, -1.3] | <0.001 |
| 50 per 100m vs. 10 per 100m | -0.4 [-0.3, -0.5] | <0.001 |
| 100 per 100m vs. 10 per 100m | -0.8 [-0.7, -0.9] | <0.001 |
| 100 per 100m vs. 50 per 100m | -0.4 [-0.5, -0.3] | <0.001 |
| **Severe allergic reactions** |  |  |
| 12 in 100k vs. 1 in 100k | -1.6 [-1.8, -1.5] | <0.001 |
| 19 in 100k vs. 1 in 100k | -2.5 [-2.7, -2.4] | <0.001 |
| 19 in 100k vs. 12 in 100k | -0.9 [-0.8, -1.0] | <0.001 |
| **Mild allergic reactions** |  |  |
| 150 in 100k vs. 10 in 100k | -1.2 [-1.3, -1.0] | <0.001 |
| 1000 in 100k vs. 10 in 100k | -2.8 [-2.9, -2.6] | <0.001 |
| 1000 in 100k vs. 150 in 100k | -1.6 [-1.8, -1.4] | <0.001 |

**Supplementary Table 3. Mean attribute importance by participant characteristics. Mean and 99% CIs**

| **Importance** | **Sensitivity** | **Out of pocket expense** | **Retention** | **Severe allergic reactions** | **Mild allergic reactions** |
| --- | --- | --- | --- | --- | --- |
| **Education** |  |  |  |  |  |
| College | 46.3 [42.6, 49.9] | 6.7 [5.7, 7.7] | 11.4 [9.7, 13.1] | 17.4 [15.5, 19.2] | 18.3 [15.9, 20.7] |
| No college | 39.8 [34.2, 45.3] | 9.5 [7.2, 11.7] | 12.3 [9.5, 15.0] | 16.0 [13.4, 18.5] | 22.5 [18.3, 26.7] |
| **Insurance** |  |  |  |  |  |
| Employer based | 44.7 [41.1, 48.2] | 7.2 [6.2, 8.3] | 11.6 [9.9, 13.2] | 17.1 [15.4, 18.9] | 19.4 [17.0, 21.8] |
| Other | 43.4 [37.0, 49.9] | 8.4 [5.8, 10.9] | 11.8 [8.8, 14.7] | 16.6 [13.6, 19.6] | 19.8 [15.6, 24.1] |
| **Employment** |  |  |  |  |  |
| Full-time | 44.2 [40.4, 48.0] | 6.9 [5.9, 7.9] | 11.7 [9.8, 13.5] | 17.2 [15.3, 19.2] | 20.0 [17.3, 22.6] |
| Other | 44.6 [39.4, 49.9] | 8.6 [6.5, 10.6] | 11.6 [9.3, 13.9] | 16.5 [14.1, 18.9] | 18.7 [15.2, 22.3] |
| **Household Income** |  |  |  |  |  |
| Less than $25,000 | 33.1 [19.9, 46.3] | 14.9 [4.6, 25.1] | 15.7 [9.0, 22.4] | 15.6 [10.7, 20.6] | 20.7 [12.5, 28.9] |
| $25,000-$49,999 | 39.9 [29.6, 50.2] | 10.2 [5.5, 14.8] | 15.8 [8.4, 23.2] | 17.5 [11.3, 23.8] | 16.5 [7.4, 25.7] |
| $50,000-$74,999 | 43.5 [31.6, 55.3] | 9.7 [5.8, 13.5] | 11.2 [6.1, 16.4] | 14.9 [10.6, 19.3] | 20.7 [10.4, 31.0] |
| $75,000-$99,999 | 39.8 [31.5, 48.0] | 7.8 [5.7, 10.0] | 12.0 [9.1, 14.9] | 16.9 [13.0, 20.9] | 23.5 [18.4, 28.6] |
| $100,000-$149,999 | 49.2 [42.6, 55.9] | 6.6 [4.9, 8.3] | 10.4 [7.6, 13.1] | 16.6 [13.1, 20.1] | 17.1 [13.1, 21.2] |
| More than $150,000 | 48.8 [43.9, 53.7] | 5.7 [4.6, 6.7] | 10.1 [7.6, 12.5] | 17.7 [14.8, 20.5] | 17.8 [14.3, 21.2] |
| Prefer not to answer | 40.7 [31.9, 49.4] | 7.1 [5.1, 9.2] | 12.7 [8.1, 17.3] | 17.3 [13.5, 21.1] | 22.2 [16.0, 28.5] |
| **Previous Allergic Reaction** |  |  |  |  |  |
| Yes | 29.4 [5.3, 53.5] | 5.7 [1.9, 9.6] | 16.9 [-1.5, 35.3] | 22.1 [8.6, 35.6] | 25.9 [12.4, 39.3] |
| No | 44.7 [41.6, 47.8] | 7.6 [6.6, 8.6] | 11.5 [10.1, 12.9] | 16.9 [15.3, 18.4] | 19.4 [17.3, 21.5] |
| *p<0.01  Attribute “importance” is the estimated average relative importance participants placed on that attribute when making product selection decisions. For each participant, attribute importance (%) is calculated as the range of their partworth utilities for that attribute, divided by the sum of the ranges for all attributes multiplied by 100 (i.e., $\frac{Specific Attribute utility range}{\sum All Attribute utility ranges} \times100$). Reported values in the first row above are the average importances across all 236 participants. | | | | | |
