# Supplementary material for: Prospective multicenter assessment of patient preferences for properties of gadolinium-based contrast media and their potential socioeconomic impact in a screening breast MRI setting
Source: Eur Radiol. 2021 May 28;31(12):9139–49. doi: 10.1007/s00330-021-07982-y (PMC8160413; doi:10.1007/s00330-021-07982-y)
Supplement: Supplementary file 2 — (PDF 1681 kb) [file 330_2021_7982_MOESM2_ESM.pdf]

Q1

Research Coordinator:

Next

Q2

Which location did the patient have their survey?

○ Blinded

Back Next

0% 100%

Q3

What is the indication for the patient's breast MRI? (If any indication other than breast cancer screening, the survey will end)

- ◯ Follow-up of probably benign findings identified on prior MRI
- ◯ New breast cancer (MRI for extent of disease)
- ◯ Evaluate response to chemotherapy
- ◯ Evaluate for residual cancer after lumpectomy with positive margins
- ◯ Metastatic cancer in the axilla without a known primary cancer
- ◯ Further evaluation of findings on mammogram or ultrasound (problem solving)
- ◯ Palpable abnormality
- ◯ Rule out implant rupture
- ◯ **Breast cancer screening (no symptoms)**
- ◯ **Breast cancer screening and rule out implant rupture (no symptoms)**

Back Next

0% 100%

Q4

Select all of the breast cancer risk factors from the list below (*High risk, + Intermediate risk):

Note: More than one risk factor can be selected, and if "none of the above" is selected the survey will end.

- ☐ *BRCA 1 mutation
- ☐ *BRCA 2 mutation
- ☐ *Other high risk gene mutations
- ☐ *The patient has not been tested but has a first-degree relative with BRCA1, BRCA2, or other high-risk gene mutation
- ☐ *History of chest radiation between ages 10 and 30
- ☐ *>20% lifetime risk of breast cancer
- ☐ **+**Personal history of breast cancer (not newly diagnosed)
- ☐ **+**History of atypical ductal hyperplasia (ADH)
- ☐ **+**History of atypical lobular hyperplasia (ALH)
- ☐ **+**History of lobular carcinoma in situ (LCIS)
- ☐ **None of the above**

Back Next

0% 100%

Q5

How old is the patient (in years)?

Back Next

0% 100%

Q6

What is the patient's gender?

- ◯ Female
- ◯ Male
- ◯ Other

Back Next

0% 100%

Q7

What is the patient's ethnicity origin (or race)?

- ◯ White
- ◯ Hispanic or Latino
- ◯ Black or African American
- ◯ American Indian or Alaska Native
- ◯ Asian/Pacific Islander
- ◯ Other

Back Next

0% 100%

Q8

Does the patient want to complete the survey? (If no, the survey will end)

- ◯ Yes
- ◯ No
- ◯ Other

Back Next

0% 100%

Q9

Please have the respondent complete the following questions:

Back Next

0% 100%

Q10

What is the highest level of education you have completed?

- ◯ Less than high school
- ◯ High school graduate (includes equivalency)
- ◯ Trade/technical/vocational training (Example: training as a construction worker, beautician, ect.)
- ◯ College graduate (Associate degree)
- ◯ College graduate (Bachelor degree)
- ◯ Graduate degree (Masters or Doctorate)
- ◯ Prefer not to answer

Back Next

0% 100%

Q11

What type of health insurance do you have?

- ○ None
- ○ Self-insured
- ○ Employer-based plan
- ○ Medicaid
- ○ Medicare
- ○ Other

Back Next

0% 100%

Q12

What is your current employment status?

- ◯ Full-time employment
- ◯ Part-time employment
- ◯ Disabled
- ◯ Unemployed
- ◯ Retired
- ◯ Prefer not to answer

Back Next

0% 100%

Q13

What was your total household income before taxes during the past 12 months?

- ◯ Less than $25,000
- ◯ $25,000 to $49,999
- ◯ $50,000 to $74,999
- ◯ $75,000 to $99,999
- ◯ $100,000 to $149,999
- ◯ More than $150,000
- ◯ Prefer not to answer

Back Next

0% 100%

Q14

Have you ever had an allergic reaction (for example, hives, rash, difficulty breathing, shock) to an imaging contrast agent?

- ◯ Yes
- ◯ No

Back Next

0% 100%

Q15

During a breast MRI, a contrast agent is given to allow your radiologist (imaging doctor) to find potential abnormalities, including breast cancer.

For each of the following options presented below, please choose which contrast agent you would prefer. There are no right or wrong answer to your preferences. **The questions will differ with the following aspects of contrast agents:**

- The likelihood your radiologist will be able to see a breast cancer if there is one present.
- The out of pocket cost of the contrast agent to you (after insurance coverage, if any).
- The amount of gadolinium from the contrast agent that stays in your brain for at least one year after the MRI. While most of the contrast agent leaves your body after the MRI, some studies have shown that a very small amount stays in the brain -- there is no scientific evidence for harm.
- The chances that the contrast agent might trigger a severe, potentially life-threatening allergic reaction (for example, shock or a narrowed airway).
- The chance that the contrast agent might trigger a nuisance allergic reaction (for example, bumps on your skin, itching, red eyes, or facial swelling).

Thank you for your time. **You will help us understand what patients value with their breast MRI.**

Back Next

0% 100%

Gadolinium_Choice1

If these were your only options, which would you choose?

1 / 15

The likelihood your radiologist will be able to see a breast cancer if there is one:

9.0 out of 10 (90%)

Out of pocket expense:

$100.00

Amount of gadolinium from the contrast agent that stays in your brain for at least one year after the MRI:
(Note: No scientific evidence exists to suggest harm)

50 molecules remain for every 100 million molecules administered (0.00005%)

The chance that a contrast agent might trigger a severe, potentially life-threatening allergic reaction:

19 in hundred-thousand (0.019%)

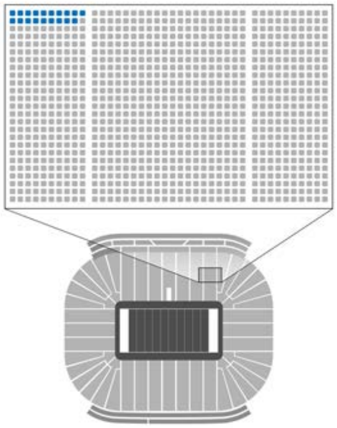

*The stadium has 100,000 seats with the filled in blue seats representing the chance of a life-threatening allergic reaction.

The likelihood your radiologist will be able to see a breast cancer if there is one:

8.0 out of 10 (80%)

Out of pocket expense:

$50.00

Amount of gadolinium from the contrast agent that stays in your brain for at least one year after the MRI:
(Note: No scientific evidence exists to suggest harm)

1 molecule remains for every 100 million molecules administered (0.000001%)

The chance that a contrast agent might trigger a severe, potentially life-threatening allergic reaction:

1 in hundred-thousand (0.001%)

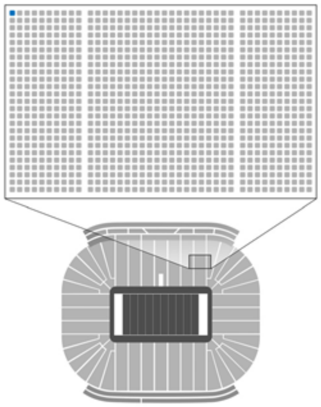

*The stadium has 100,000 seats with the filled in blue seats representing the chance of a life-threatening allergic reaction.

The chances that a contrast agent might trigger a nuisance allergic reaction:

1000 in hundred-thousand (1%)

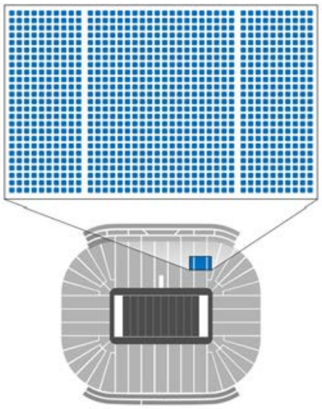

*The stadium has 100,000 seats with the filled in blue seats representing the chance of a nuisance allergic reaction.

Select

The chances that a contrast agent might trigger a nuisance allergic reaction:

150 in hundred-thousand (0.15%)

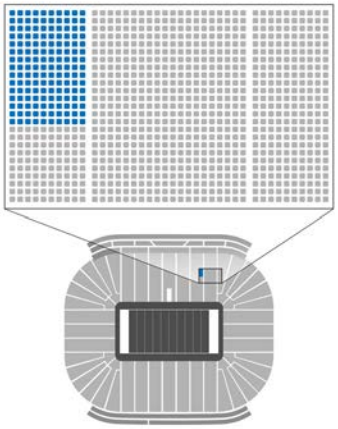

*The stadium has 100,000 seats with the filled in blue seats representing the chance of a nuisance allergic reaction.

Select

Back Next

0% 100%

Gadolinium_Choice2

If these were your only options, which would you choose?

2 / 15

The likelihood your radiologist will be able to see a breast cancer if there is one:

9.5 out of 10 (95%)

Out of pocket expense:

$25.00

Amount of gadolinium from the contrast agent that stays in your brain for at least one year after the MRI:
(Note: No scientific evidence exists to suggest harm)

10 molecules remain for every 100 million molecules administered (0.00001%)

The chance that a contrast agent might trigger a severe, potentially life-threatening allergic reaction:

12 in hundred-thousand (0.012%)

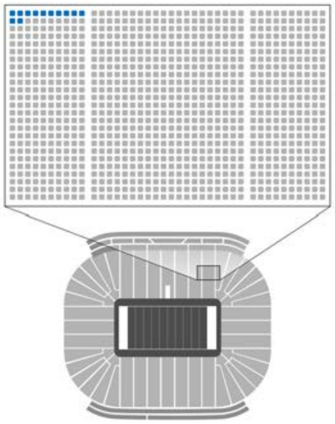

*The stadium has 100,000 seats with the filled in blue seats representing the chance of a life-threatening allergic reaction.

The likelihood your radiologist will be able to see a breast cancer if there is one:

8.5 out of 10 (85%)

Out of pocket expense:

$25.00

Amount of gadolinium from the contrast agent that stays in your brain for at least one year after the MRI:
(Note: No scientific evidence exists to suggest harm)

100 molecule remains for every 100 million molecules administered (0.0001%)

The chance that a contrast agent might trigger a severe, potentially life-threatening allergic reaction:

1 in hundred-thousand (0.001%)

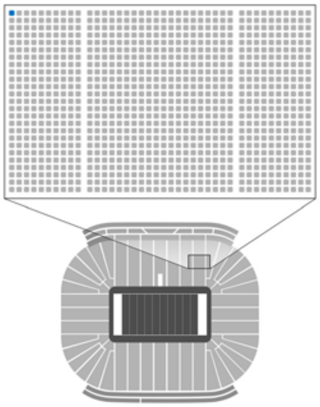

*The stadium has 100,000 seats with the filled in blue seats representing the chance of a life-threatening allergic reaction.

The chances that a contrast agent might trigger a nuisance allergic reaction:

10 in hundred-thousand (0.01%)

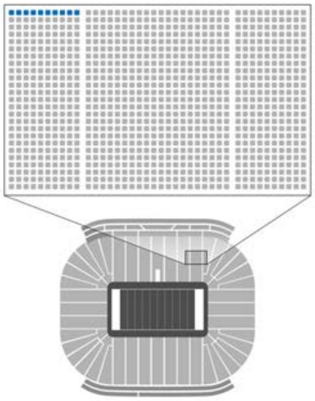

*The stadium has 100,000 seats with the filled in blue seats representing the chance of a nuisance allergic reaction.

Select

The chances that a contrast agent might trigger a nuisance allergic reaction:

10 in hundred-thousand (0.01%)

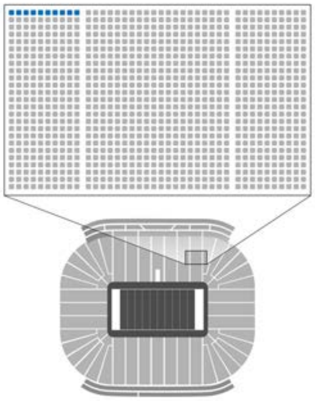

*The stadium has 100,000 seats with the filled in blue seats representing the chance of a nuisance allergic reaction.

Select

Back Next

0% 100%

Gadolinium_Choice3

If these were your only options, which would you choose?

3 / 15

The likelihood your radiologist will be able to see a breast cancer if there is one:

9.5 out of 10 (95%)

Out of pocket expense:

$25.00

Amount of gadolinium from the contrast agent that stays in your brain for at least one year after the MRI:
(Note: No scientific evidence exists to suggest harm)

50 molecules remain for every 100 million molecules administered (0.00005%)

The chance that a contrast agent might trigger a severe, potentially life-threatening allergic reaction:

12 in hundred-thousand (0.012%)

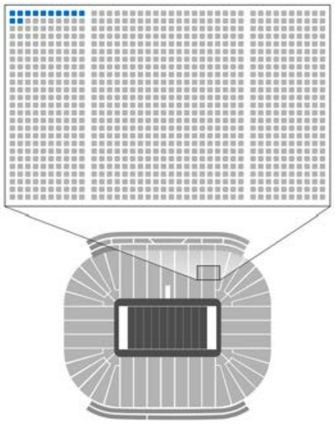

*The stadium has 100,000 seats with the filled in blue seats representing the chance of a life-threatening allergic reaction.

The likelihood your radiologist will be able to see a breast cancer if there is one:

8.5 out of 10 (85%)

Out of pocket expense:

$50.00

Amount of gadolinium from the contrast agent that stays in your brain for at least one year after the MRI:
(Note: No scientific evidence exists to suggest harm)

10 molecules remain for every 100 million molecules administered (0.00001%)

The chance that a contrast agent might trigger a severe, potentially life-threatening allergic reaction:

19 in hundred-thousand (0.019%)

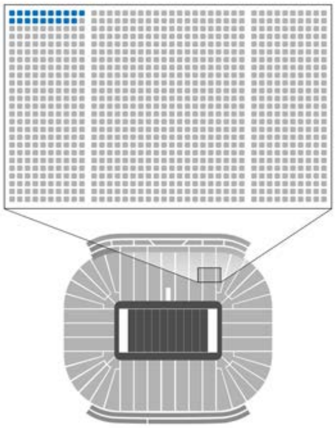

*The stadium has 100,000 seats with the filled in blue seats representing the chance of a life-threatening allergic reaction.

The chances that a contrast agent might trigger a nuisance allergic reaction:

1000 in hundred-thousand (1%)

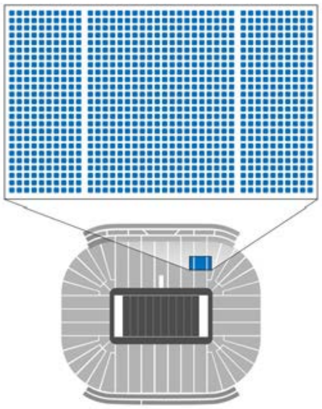

*The stadium has 100,000 seats with the filled in blue seats representing the chance of a nuisance allergic reaction.

Select

The chances that a contrast agent might trigger a nuisance allergic reaction:

150 in hundred-thousand (0.15%)

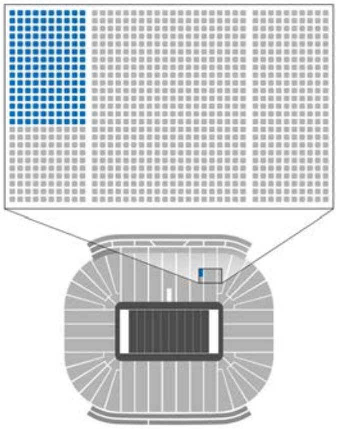

*The stadium has 100,000 seats with the filled in blue seats representing the chance of a nuisance allergic reaction.

Select

Back Next

0% 100%

Gadolinium_Choice4

If these were your only options, which would you choose?

4 / 15

The likelihood your radiologist will be able to see a breast cancer if there is one:

8.0 out of 10 (80%)

Out of pocket expense:

$100.00

Amount of gadolinium from the contrast agent that stays in your brain for at least one year after the MRI:
(Note: No scientific evidence exists to suggest harm)

1 molecule remains for every 100 million molecules administered (0.000001%)

The chance that a contrast agent might trigger a severe, potentially life-threatening allergic reaction:

1 in hundred-thousand (0.001%)

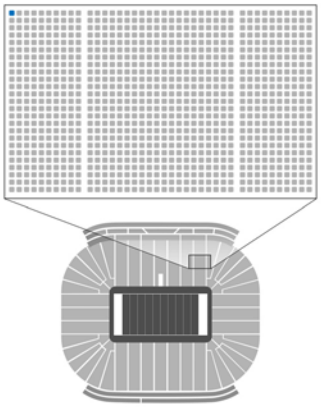

*The stadium has 100,000 seats with the filled in blue seats representing the chance of a life-threatening allergic reaction.

The chances that a contrast agent might trigger

The likelihood your radiologist will be able to see a breast cancer if there is one:

9.0 out of 10 (90%)

Out of pocket expense:

$25.00

Amount of gadolinium from the contrast agent that stays in your brain for at least one year after the MRI:
(Note: No scientific evidence exists to suggest harm)

100 molecule remains for every 100 million molecules administered (0.0001%)

The chance that a contrast agent might trigger a severe, potentially life-threatening allergic reaction:

1 in hundred-thousand (0.001%)

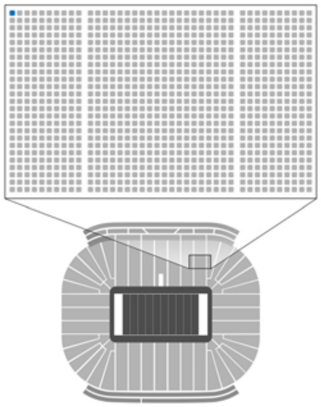

*The stadium has 100,000 seats with the filled in blue seats representing the chance of a life-threatening allergic reaction.

The chances that a contrast agent might trigger

a nuisance allergic reaction:

10 in hundred-thousand (0.01%)

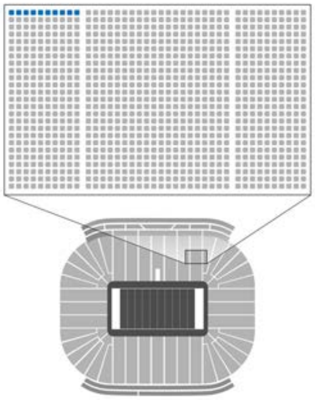

*The stadium has 100,000 seats with the filled in blue seats representing the chance of a nuisance allergic reaction.

Select

a nuisance allergic reaction:

10 in hundred-thousand (0.01%)

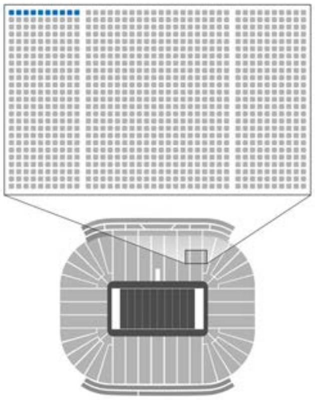

*The stadium has 100,000 seats with the filled in blue seats representing the chance of a nuisance allergic reaction.

Select

Back Next

0% 100%

Gadolinium_Choice5

If these were your only options, which would you choose?

5 / 15

The likelihood your radiologist will be able to see a breast cancer if there is one:

8.5 out of 10 (85%)

Out of pocket expense:

$100.00

Amount of gadolinium from the contrast agent that stays in your brain for at least one year after the MRI:
(Note: No scientific evidence exists to suggest harm)

10 molecules remain for every 100 million molecules administered (0.00001%)

The chance that a contrast agent might trigger a severe, potentially life-threatening allergic reaction:

1 in hundred-thousand (0.001%)

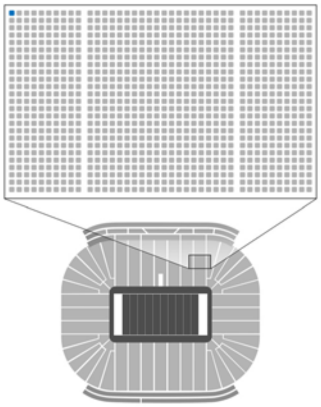

*The stadium has 100,000 seats with the filled in blue seats representing the chance of a life-threatening allergic reaction.

The likelihood your radiologist will be able to see a breast cancer if there is one:

9.0 out of 10 (90%)

Out of pocket expense:

$50.00

Amount of gadolinium from the contrast agent that stays in your brain for at least one year after the MRI:
(Note: No scientific evidence exists to suggest harm)

100 molecule remains for every 100 million molecules administered (0.0001%)

The chance that a contrast agent might trigger a severe, potentially life-threatening allergic reaction:

12 in hundred-thousand (0.012%)

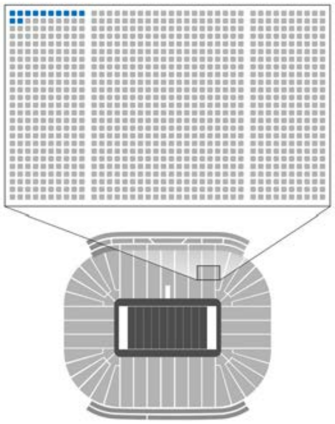

*The stadium has 100,000 seats with the filled in blue seats representing the chance of a life-threatening allergic reaction.

The chances that a contrast agent might trigger a nuisance allergic reaction:

10 in hundred-thousand (0.01%)

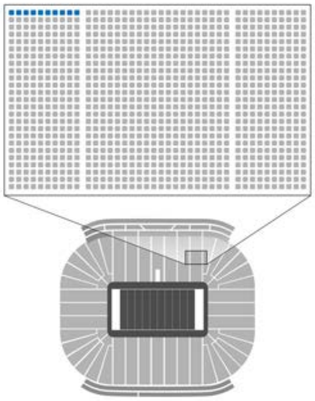

*The stadium has 100,000 seats with the filled in blue seats representing the chance of a nuisance allergic reaction.

Select

The chances that a contrast agent might trigger a nuisance allergic reaction:

1000 in hundred-thousand (1%)

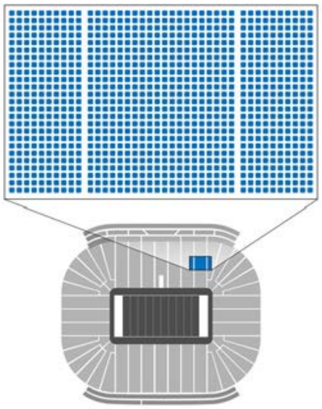

*The stadium has 100,000 seats with the filled in blue seats representing the chance of a nuisance allergic reaction.

Select

Back Next

0% 100%

Gadolinium_Choice6

If these were your only options, which would you choose?

6 / 15

The likelihood your radiologist will be able to see a breast cancer if there is one:

8.0 out of 10 (80%)

Out of pocket expense:

$25.00

Amount of gadolinium from the contrast agent that stays in your brain for at least one year after the MRI:
(Note: No scientific evidence exists to suggest harm)

1 molecule remains for every 100 million molecules administered (0.000001%)

The chance that a contrast agent might trigger a severe, potentially life-threatening allergic reaction:

19 in hundred-thousand (0.019%)

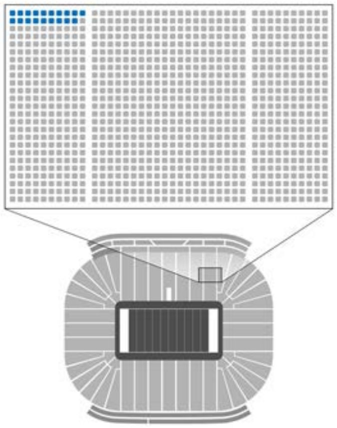

*The stadium has 100,000 seats with the filled in blue seats representing the chance of a life-threatening allergic reaction.

The likelihood your radiologist will be able to see a breast cancer if there is one:

9.5 out of 10 (95%)

Out of pocket expense:

$25.00

Amount of gadolinium from the contrast agent that stays in your brain for at least one year after the MRI:
(Note: No scientific evidence exists to suggest harm)

50 molecules remain for every 100 million molecules administered (0.00005%)

The chance that a contrast agent might trigger a severe, potentially life-threatening allergic reaction:

1 in hundred-thousand (0.001%)

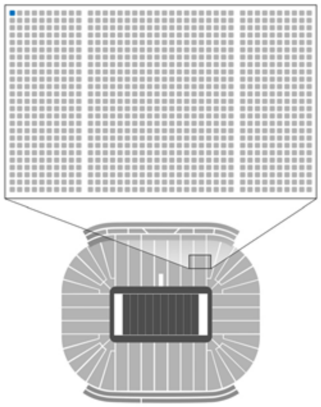

*The stadium has 100,000 seats with the filled in blue seats representing the chance of a life-threatening allergic reaction.

The chances that a contrast agent might trigger a nuisance allergic reaction:

150 in hundred-thousand (0.15%)

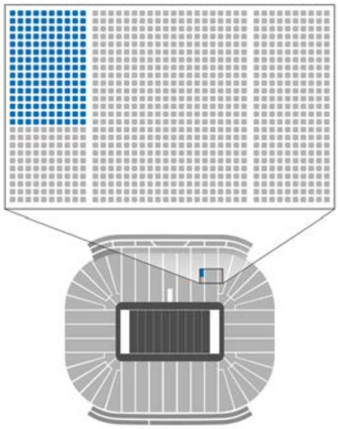

*The stadium has 100,000 seats with the filled in blue seats representing the chance of a nuisance allergic reaction.

Select

The chances that a contrast agent might trigger a nuisance allergic reaction:

10 in hundred-thousand (0.01%)

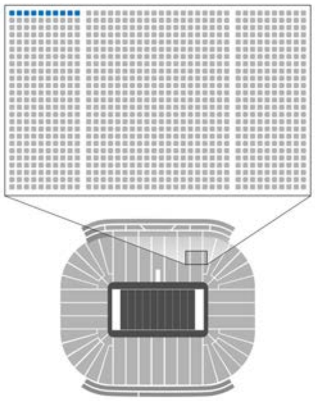

*The stadium has 100,000 seats with the filled in blue seats representing the chance of a nuisance allergic reaction.

Select

Back Next

0% 100%

Gadolinium_Choice7

If these were your only options, which would you choose?

7 / 15

The likelihood your radiologist will be able to see a breast cancer if there is one:

8.5 out of 10 (85%)

Out of pocket expense:

$100.00

Amount of gadolinium from the contrast agent that stays in your brain for at least one year after the MRI:
(Note: No scientific evidence exists to suggest harm)

10 molecules remain for every 100 million molecules administered (0.00001%)

The chance that a contrast agent might trigger a severe, potentially life-threatening allergic reaction:

19 in hundred-thousand (0.019%)

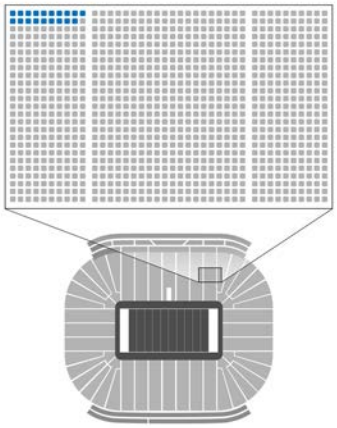

*The stadium has 100,000 seats with the filled in blue seats representing the chance of a life-threatening allergic reaction.

The likelihood your radiologist will be able to see a breast cancer if there is one:

8.0 out of 10 (80%)

Out of pocket expense:

$50.00

Amount of gadolinium from the contrast agent that stays in your brain for at least one year after the MRI:
(Note: No scientific evidence exists to suggest harm)

50 molecules remain for every 100 million molecules administered (0.00005%)

The chance that a contrast agent might trigger a severe, potentially life-threatening allergic reaction:

12 in hundred-thousand (0.012%)

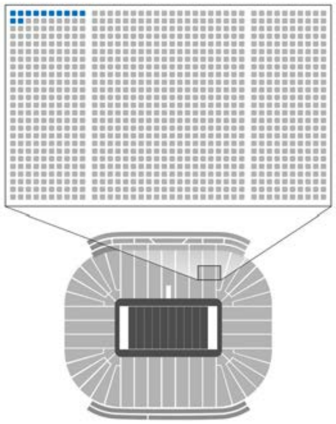

*The stadium has 100,000 seats with the filled in blue seats representing the chance of a life-threatening allergic reaction.

The chances that a contrast agent might trigger a nuisance allergic reaction:

1000 in hundred-thousand (1%)

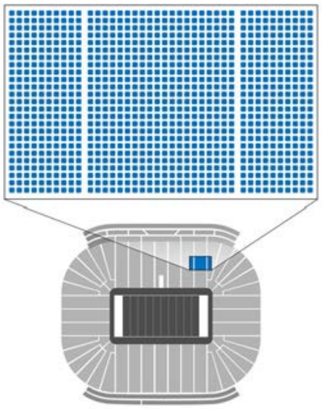

*The stadium has 100,000 seats with the filled in blue seats representing the chance of a nuisance allergic reaction.

Select

The chances that a contrast agent might trigger a nuisance allergic reaction:

10 in hundred-thousand (0.01%)

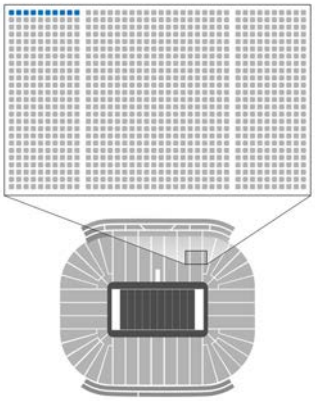

*The stadium has 100,000 seats with the filled in blue seats representing the chance of a nuisance allergic reaction.

Select

Back Next

0% 100%

Gadolinium_Choice8

If these were your only options, which would you choose?

8 / 15

The likelihood your radiologist will be able to see a breast cancer if there is one:

9.5 out of 10 (95%)

Out of pocket expense:

$25.00

Amount of gadolinium from the contrast agent that stays in your brain for at least one year after the MRI:
(Note: No scientific evidence exists to suggest harm)

1 molecule remains for every 100 million molecules administered (0.000001%)

The chance that a contrast agent might trigger a severe, potentially life-threatening allergic reaction:

1 in hundred-thousand (0.001%)

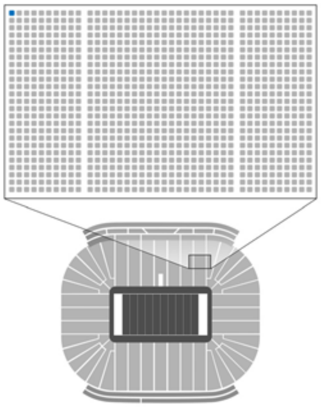

*The stadium has 100,000 seats with the filled in blue seats representing the chance of a life-threatening allergic reaction.

The chances that a contrast agent might trigger

The likelihood your radiologist will be able to see a breast cancer if there is one:

9.0 out of 10 (90%)

Out of pocket expense:

$25.00

Amount of gadolinium from the contrast agent that stays in your brain for at least one year after the MRI:
(Note: No scientific evidence exists to suggest harm)

100 molecule remains for every 100 million molecules administered (0.0001%)

The chance that a contrast agent might trigger a severe, potentially life-threatening allergic reaction:

1 in hundred-thousand (0.001%)

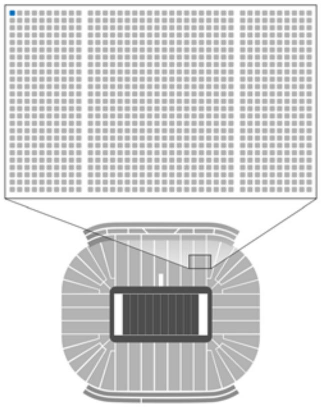

*The stadium has 100,000 seats with the filled in blue seats representing the chance of a life-threatening allergic reaction.

The chances that a contrast agent might trigger

a nuisance allergic reaction:

150 in hundred-thousand (0.15%)

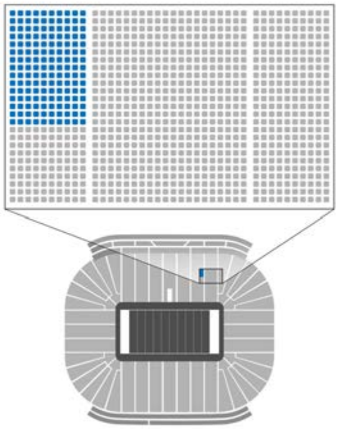

*The stadium has 100,000 seats with the filled in blue seats representing the chance of a nuisance allergic reaction.

Select

a nuisance allergic reaction:

10 in hundred-thousand (0.01%)

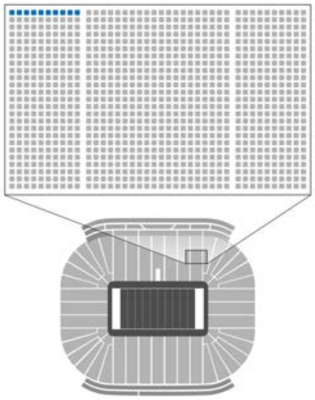

*The stadium has 100,000 seats with the filled in blue seats representing the chance of a nuisance allergic reaction.

Select

Back Next

0% 100%

Gadolinium_Choice9

If these were your only options, which would you choose?

9 / 15

The likelihood your radiologist will be able to see a breast cancer if there is one:

9.0 out of 10 (90%)

Out of pocket expense:

$50.00

Amount of gadolinium from the contrast agent that stays in your brain for at least one year after the MRI:
(Note: No scientific evidence exists to suggest harm)

10 molecules remain for every 100 million molecules administered (0.00001%)

The chance that a contrast agent might trigger a severe, potentially life-threatening allergic reaction:

1 in hundred-thousand (0.001%)

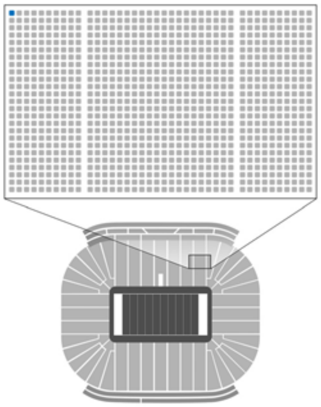

*The stadium has 100,000 seats with the filled in blue seats representing the chance of a life-threatening allergic reaction.

The likelihood your radiologist will be able to see a breast cancer if there is one:

8.0 out of 10 (80%)

Out of pocket expense:

$25.00

Amount of gadolinium from the contrast agent that stays in your brain for at least one year after the MRI:
(Note: No scientific evidence exists to suggest harm)

50 molecules remain for every 100 million molecules administered (0.00005%)

The chance that a contrast agent might trigger a severe, potentially life-threatening allergic reaction:

12 in hundred-thousand (0.012%)

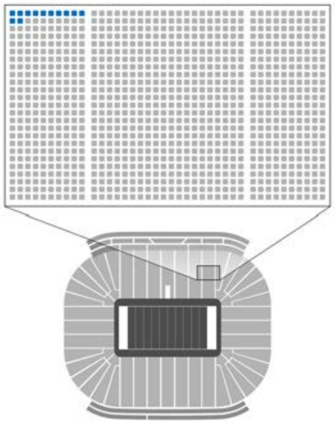

*The stadium has 100,000 seats with the filled in blue seats representing the chance of a life-threatening allergic reaction.

The chances that a contrast agent might trigger a nuisance allergic reaction:

10 in hundred-thousand (0.01%)

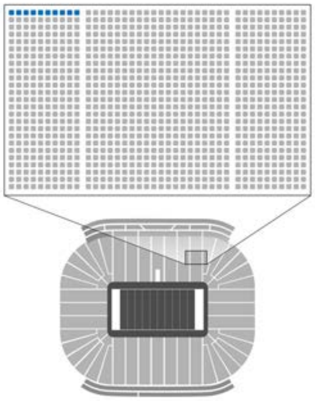

*The stadium has 100,000 seats with the filled in blue seats representing the chance of a nuisance allergic reaction.

Select

The chances that a contrast agent might trigger a nuisance allergic reaction:

1000 in hundred-thousand (1%)

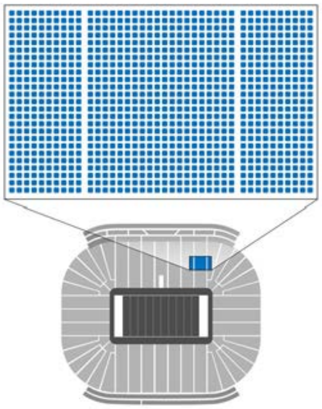

*The stadium has 100,000 seats with the filled in blue seats representing the chance of a nuisance allergic reaction.

Select

Back Next

0% 100%

Gadolinium_Choice10

If these were your only options, which would you choose?

10 / 15

The likelihood your radiologist will be able to see a breast cancer if there is one:

8.5 out of 10 (85%)

Out of pocket expense:

$100.00

Amount of gadolinium from the contrast agent that stays in your brain for at least one year after the MRI:
(Note: No scientific evidence exists to suggest harm)

100 molecule remains for every 100 million molecules administered (0.0001%)

The chance that a contrast agent might trigger a severe, potentially life-threatening allergic reaction:

19 in hundred-thousand (0.019%)

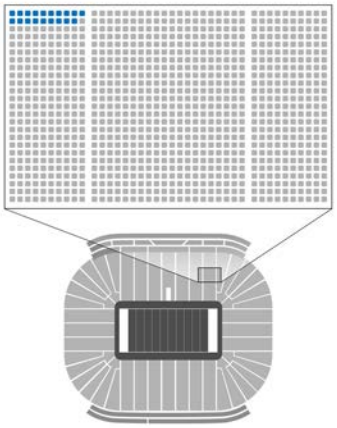

*The stadium has 100,000 seats with the filled in blue seats representing the chance of a life-threatening allergic reaction.

The likelihood your radiologist will be able to see a breast cancer if there is one:

9.0 out of 10 (90%)

Out of pocket expense:

$25.00

Amount of gadolinium from the contrast agent that stays in your brain for at least one year after the MRI:
(Note: No scientific evidence exists to suggest harm)

10 molecules remain for every 100 million molecules administered (0.00001%)

The chance that a contrast agent might trigger a severe, potentially life-threatening allergic reaction:

19 in hundred-thousand (0.019%)

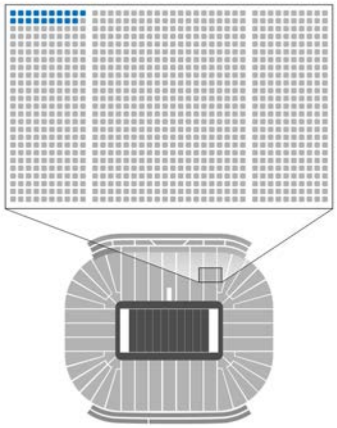

*The stadium has 100,000 seats with the filled in blue seats representing the chance of a life-threatening allergic reaction.

The chances that a contrast agent might trigger a nuisance allergic reaction:

150 in hundred-thousand (0.15%)

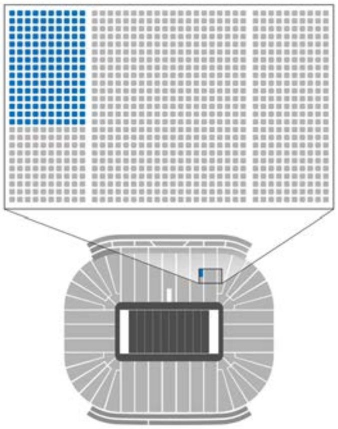

*The stadium has 100,000 seats with the filled in blue seats representing the chance of a nuisance allergic reaction.

Select

The chances that a contrast agent might trigger a nuisance allergic reaction:

1000 in hundred-thousand (1%)

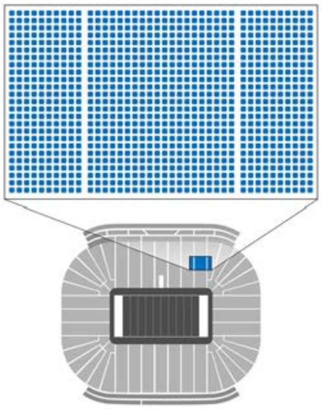

*The stadium has 100,000 seats with the filled in blue seats representing the chance of a nuisance allergic reaction.

Select

Back Next

0% 100%

Gadolinium_Choice11

If these were your only options, which would you choose?

11 / 15

The likelihood your radiologist will be able to see a breast cancer if there is one:

8.5 out of 10 (85%)

Out of pocket expense:

$50.00

Amount of gadolinium from the contrast agent that stays in your brain for at least one year after the MRI:
(Note: No scientific evidence exists to suggest harm)

1 molecule remains for every 100 million molecules administered (0.000001%)

The chance that a contrast agent might trigger a severe, potentially life-threatening allergic reaction:

1 in hundred-thousand (0.001%)

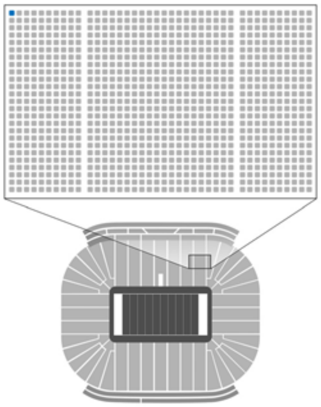

*The stadium has 100,000 seats with the filled in blue seats representing the chance of a life-threatening allergic reaction.

The likelihood your radiologist will be able to see a breast cancer if there is one:

9.5 out of 10 (95%)

Out of pocket expense:

$100.00

Amount of gadolinium from the contrast agent that stays in your brain for at least one year after the MRI:
(Note: No scientific evidence exists to suggest harm)

100 molecule remains for every 100 million molecules administered (0.0001%)

The chance that a contrast agent might trigger a severe, potentially life-threatening allergic reaction:

12 in hundred-thousand (0.012%)

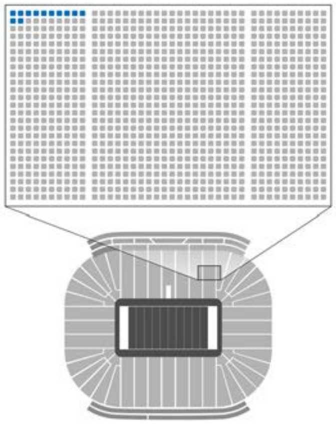

*The stadium has 100,000 seats with the filled in blue seats representing the chance of a life-threatening allergic reaction.

The chances that a contrast agent might trigger a nuisance allergic reaction:

10 in hundred-thousand (0.01%)

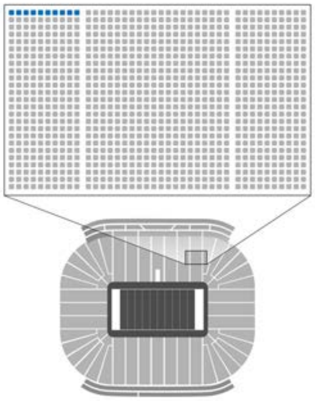

*The stadium has 100,000 seats with the filled in blue seats representing the chance of a nuisance allergic reaction.

Select

The chances that a contrast agent might trigger a nuisance allergic reaction:

150 in hundred-thousand (0.15%)

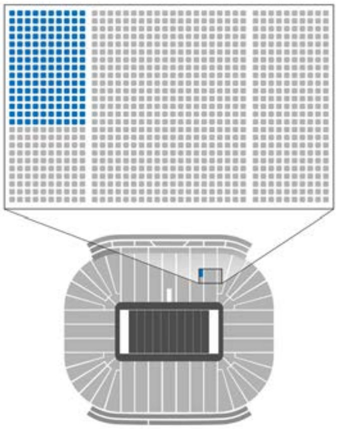

*The stadium has 100,000 seats with the filled in blue seats representing the chance of a nuisance allergic reaction.

Select

Back Next

0% 100%

Gadolinium_Choice12

If these were your only options, which would you choose?

12 / 15

The likelihood your radiologist will be able to see a breast cancer if there is one:

8.0 out of 10 (80%)

Out of pocket expense:

$25.00

Amount of gadolinium from the contrast agent that stays in your brain for at least one year after the MRI:
(Note: No scientific evidence exists to suggest harm)

50 molecules remain for every 100 million molecules administered (0.00005%)

The chance that a contrast agent might trigger a severe, potentially life-threatening allergic reaction:

1 in hundred-thousand (0.001%)

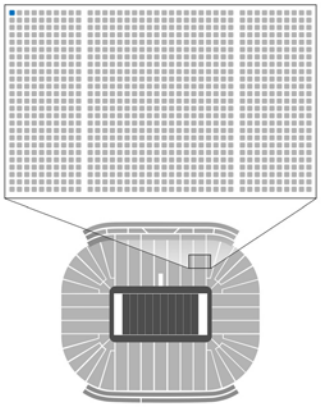

*The stadium has 100,000 seats with the filled in blue seats representing the chance of a life-threatening allergic reaction.

The likelihood your radiologist will be able to see a breast cancer if there is one:

8.0 out of 10 (80%)

Out of pocket expense:

$100.00

Amount of gadolinium from the contrast agent that stays in your brain for at least one year after the MRI:
(Note: No scientific evidence exists to suggest harm)

10 molecules remain for every 100 million molecules administered (0.00001%)

The chance that a contrast agent might trigger a severe, potentially life-threatening allergic reaction:

19 in hundred-thousand (0.019%)

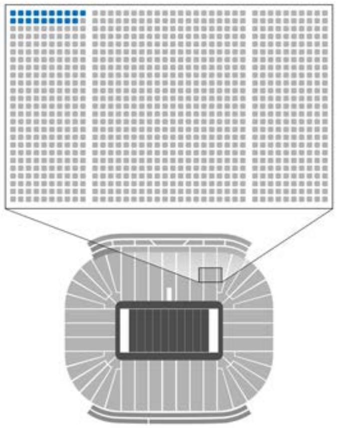

*The stadium has 100,000 seats with the filled in blue seats representing the chance of a life-threatening allergic reaction.

The chances that a contrast agent might trigger a nuisance allergic reaction:

10 in hundred-thousand (0.01%)

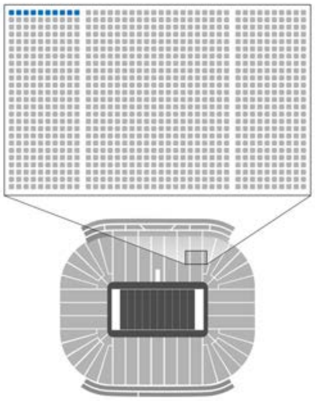

*The stadium has 100,000 seats with the filled in blue seats representing the chance of a nuisance allergic reaction.

Select

The chances that a contrast agent might trigger a nuisance allergic reaction:

150 in hundred-thousand (0.15%)

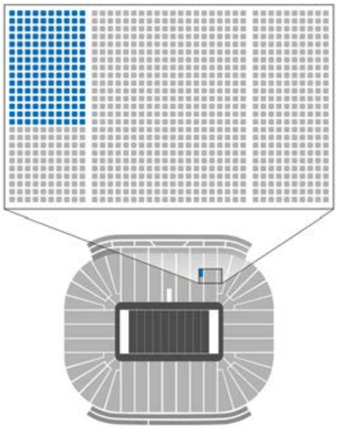

*The stadium has 100,000 seats with the filled in blue seats representing the chance of a nuisance allergic reaction.

Select

Back Next

0% 100%

Gadolinium_Choice13

If these were your only options, which would you choose?

13 / 15

The likelihood your radiologist will be able to see a breast cancer if there is one:

8.5 out of 10 (85%)

Out of pocket expense:

$50.00

Amount of gadolinium from the contrast agent that stays in your brain for at least one year after the MRI:
(Note: No scientific evidence exists to suggest harm)

100 molecule remains for every 100 million molecules administered (0.0001%)

The chance that a contrast agent might trigger a severe, potentially life-threatening allergic reaction:

12 in hundred-thousand (0.012%)

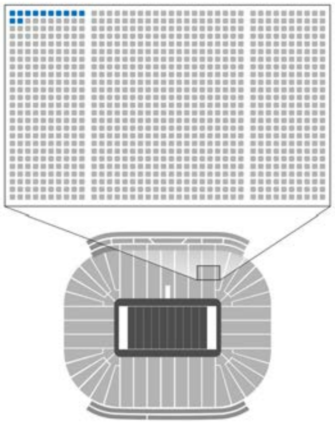

*The stadium has 100,000 seats with the filled in blue seats representing the chance of a life-threatening allergic reaction.

The likelihood your radiologist will be able to see a breast cancer if there is one:

8.0 out of 10 (80%)

Out of pocket expense:

$100.00

Amount of gadolinium from the contrast agent that stays in your brain for at least one year after the MRI:
(Note: No scientific evidence exists to suggest harm)

50 molecules remain for every 100 million molecules administered (0.00005%)

The chance that a contrast agent might trigger a severe, potentially life-threatening allergic reaction:

19 in hundred-thousand (0.019%)

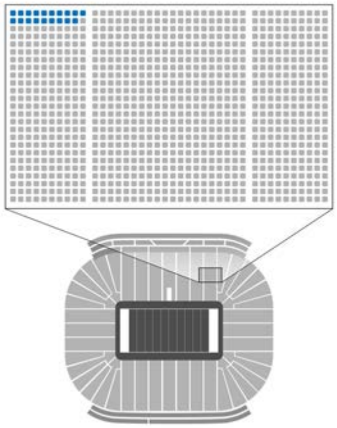

*The stadium has 100,000 seats with the filled in blue seats representing the chance of a life-threatening allergic reaction.

The chances that a contrast agent might trigger a nuisance allergic reaction:

1000 in hundred-thousand (1%)

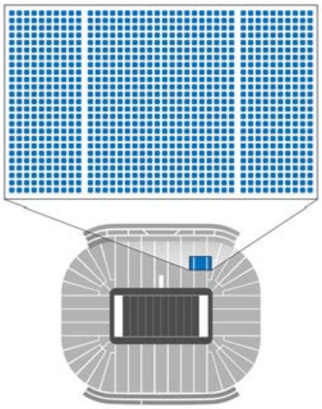

*The stadium has 100,000 seats with the filled in blue seats representing the chance of a nuisance allergic reaction.

Select

The chances that a contrast agent might trigger a nuisance allergic reaction:

10 in hundred-thousand (0.01%)

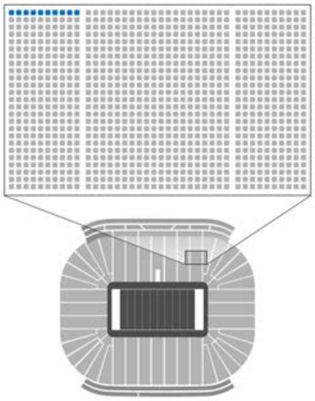

*The stadium has 100,000 seats with the filled in blue seats representing the chance of a nuisance allergic reaction.

Select

Back Next

0% 100%

Gadolinium_Choice14

If these were your only options, which would you choose?

14 / 15

The likelihood your radiologist will be able to see a breast cancer if there is one:

9.0 out of 10 (90%)

Out of pocket expense:

$25.00

Amount of gadolinium from the contrast agent that stays in your brain for at least one year after the MRI:
(Note: No scientific evidence exists to suggest harm)

50 molecules remain for every 100 million molecules administered (0.00005%)

The chance that a contrast agent might trigger a severe, potentially life-threatening allergic reaction:

1 in hundred-thousand (0.001%)

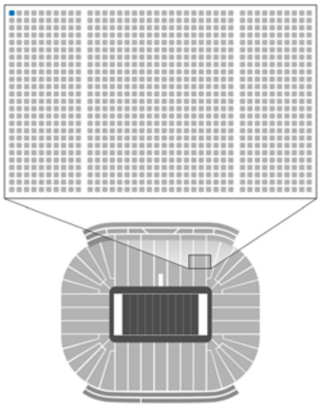

*The stadium has 100,000 seats with the filled in blue seats representing the chance of a life-threatening allergic reaction.

The chances that a contrast agent might trigger

The likelihood your radiologist will be able to see a breast cancer if there is one:

9.5 out of 10 (95%)

Out of pocket expense:

$50.00

Amount of gadolinium from the contrast agent that stays in your brain for at least one year after the MRI:
(Note: No scientific evidence exists to suggest harm)

10 molecules remain for every 100 million molecules administered (0.00001%)

The chance that a contrast agent might trigger a severe, potentially life-threatening allergic reaction:

1 in hundred-thousand (0.001%)

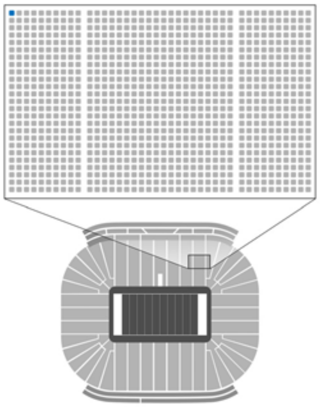

*The stadium has 100,000 seats with the filled in blue seats representing the chance of a life-threatening allergic reaction.

The chances that a contrast agent might trigger

a nuisance allergic reaction:

10 in hundred-thousand (0.01%)

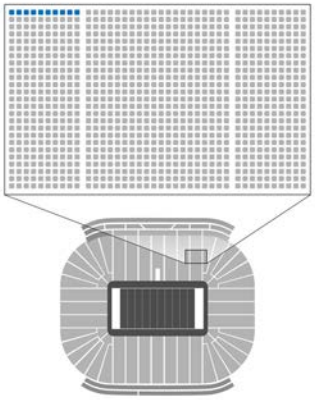

*The stadium has 100,000 seats with the filled in blue seats representing the chance of a nuisance allergic reaction.

Select

a nuisance allergic reaction:

150 in hundred-thousand (0.15%)

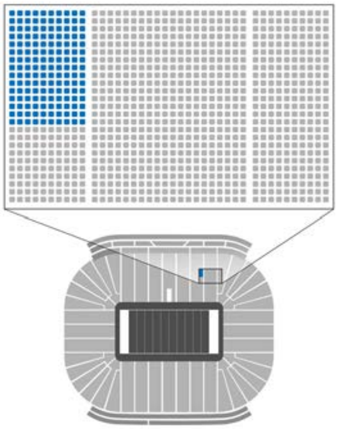

*The stadium has 100,000 seats with the filled in blue seats representing the chance of a nuisance allergic reaction.

Select

Back Next

0% 100%

Gadolinium_Choice15

If these were your only options, which would you choose?

15 / 15

The likelihood your radiologist will be able to see a breast cancer if there is one:

9.0 out of 10 (90%)

Out of pocket expense:

$25.00

Amount of gadolinium from the contrast agent that stays in your brain for at least one year after the MRI:
(Note: No scientific evidence exists to suggest harm)

100 molecule remains for every 100 million molecules administered (0.0001%)

The chance that a contrast agent might trigger a severe, potentially life-threatening allergic reaction:

12 in hundred-thousand (0.012%)

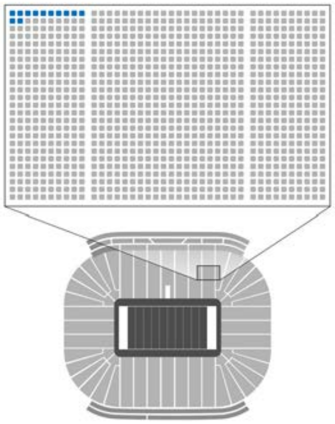

*The stadium has 100,000 seats with the filled in blue seats representing the chance of a life-threatening allergic reaction.

The likelihood your radiologist will be able to see a breast cancer if there is one:

8.5 out of 10 (85%)

Out of pocket expense:

$25.00

Amount of gadolinium from the contrast agent that stays in your brain for at least one year after the MRI:
(Note: No scientific evidence exists to suggest harm)

1 molecule remains for every 100 million molecules administered (0.000001%)

The chance that a contrast agent might trigger a severe, potentially life-threatening allergic reaction:

1 in hundred-thousand (0.001%)

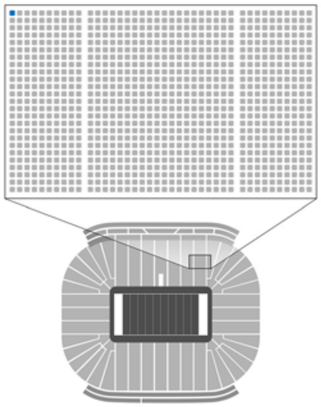

*The stadium has 100,000 seats with the filled in blue seats representing the chance of a life-threatening allergic reaction.

The chances that a contrast agent might trigger a nuisance allergic reaction:

1000 in hundred-thousand (1%)

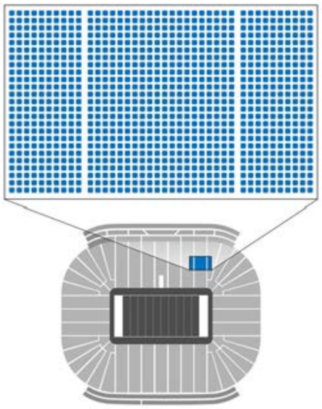

*The stadium has 100,000 seats with the filled in blue seats representing the chance of a nuisance allergic reaction.

Select

The chances that a contrast agent might trigger a nuisance allergic reaction:

10 in hundred-thousand (0.01%)

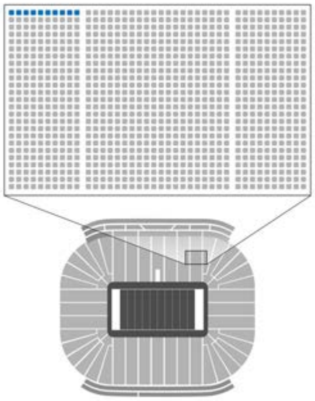

*The stadium has 100,000 seats with the filled in blue seats representing the chance of a nuisance allergic reaction.

Select

Back Next

0% 100%

Q16

You are finished. Thank you for taking this survey. We appreciate your feedback.

0% 100%
